# Regulation of plasma von Willebrand factor

**DOI:** 10.12688/f1000research.13056.1

**Published:** 2018-01-23

**Authors:** Karl C Desch

**Affiliations:** 1 Department of Pediatrics and Communicable Diseases, University of Michigan, Ann Arbor, Michigan, USA

**Keywords:** von Willebrand factor, coagulation factor VIII, plasma von Willebrand factor levels, hemostasis

## Abstract

Von Willebrand factor (VWF) is a multimeric plasma glycoprotein that plays a central role in the initiation of blood coagulation. Through interactions between its specific functional domains, the vascular wall, coagulation factor VIII, and platelet receptors, VWF maintains hemostasis by binding to platelets and delivering factor VIII to the sites of vascular injury. In the healthy human population, plasma VWF levels vary widely. The important role of VWF is illustrated by individuals at the extremes of the normal distribution of plasma VWF concentrations where individuals with low VWF levels are more likely to present with mucocutaneous bleeding. Conversely, people with high VWF levels are at higher risk for venous thromboembolic disease, stroke, and coronary artery disease. This report will summarize recent advances in our understanding of environmental influences and the genetic control of VWF plasma variation in healthy and symptomatic populations and will also highlight the unanswered questions that are currently driving this field of study.

## Introduction

Genetic studies of plasma von Willebrand factor (VWF) variation are a paradigm for the genetic regulation of quantitative traits. VWF is a fascinating trait to study because it has a wide distribution in healthy populations. Severe medical disorders, like venous thromboembolic disease (VTE) and von Willebrand disease (VWD), are manifested in people with abnormal VWF concentrations or function ^1^. Investigations of the genetic determinants of plasma VWF, unlike many complex genetic traits, have been successful at explaining a large portion of the heritability of this trait. While other important medical diseases such as type II diabetes and coronary artery disease rely on descriptive clinical phenotyping and arose relatively recently in human populations, plasma VWF levels are an easily measured proxy for bleeding or thrombosis risk, which are two traits with likely high selective pressures in human evolution. These pressures may have led to a genetic architecture where a relatively limited number of genes determine plasma VWF levels compared with several hundreds of genes that have been associated with other complex traits, such as human height variation ^2^. Additionally, VWF levels are highly heritable; recent estimates of about 65% suggest that most of the variation of VWF levels in a healthy population is caused by genetic factors ^3^. The clinical relevance, easily measurable nature, and high heritability of VWF levels make this an ideal trait to investigate with modern genome-wide tools. This report will review recent genome-wide studies of VWF variation and the functional studies that have followed to better understand the genetic determinants of VWF levels. Questions remaining at this time center on the role played by rare human genetic variants outside of the VWF locus in the regulation of VWF levels. Likewise, investigators are extremely interested to determine what particular genes or genetic variants contribute to an individual’s risk for bleeding or thrombotic disease.

## Environmental covariates

Complex genetic traits, such as plasma VWF levels, are determined by both genetic and environmental factors as well as interactions between these factors. For optimal signal-to-noise ratios, environmental factors must be controlled for in genetic association studies. There are several well-described environmental influences on plasma VWF; the largest of these is age, as VWF concentrations increase over an individual’s lifetime. This correlation was made by sampling populations of different ages and documenting a significant correlation between age and VWF levels. A well-powered longitudinal study measuring individuals’ VWF levels over years would be more definitive. Mechanistically, the increase in VWF with age seems to be caused by both an increased synthesis/secretion rate and a decreased clearance from the circulation ^4^. However, it is not clear what environmental or genetic factors cause the increased synthesis/secretion and decreased clearance of VWF with age. Increased endothelial activation (increases VWF synthesis/secretion) and decreased expression or activity of VWF clearance receptors are suspected ^4^. Diurnal, monthly, or seasonal variation may also add error to the interpretation of a one-time measurement of plasma VWF. A UK study of about 9,000 individuals who were 45 years old suggested that VWF does have statistically significant diurnal and seasonal variation but that the overall size of the variation was small (about 2%) ^5^. Additionally, studies of VWF variation with menstrual cycles have had discrepant results but suggest that VWF levels are lowest during the menstrual and early follicular stages ^6^. Use of oral contraceptive pills is a risk factor for VTE, and they can be used as therapeutic agents for women with menorrhagia and VWD. However, it remains unclear whether the use of modern, lower-estrogen oral contraceptive pills increases VTE risk through elevated VWF levels ^7^.

VWF is an acute-phase reactant. Therefore, in stressful states such as pregnancy, labor, acute infection, or surgery, VWF levels rise through increased secretion rates. Interestingly, cord blood levels of VWF are higher in neonates born to laboring mothers compared with those born by elective cesarean section, suggesting that the acute-phase response also functions in the perinatal period ^8^. Additionally, strenuous exercise is a well-documented environmental factor, causing a twofold elevation of plasma VWF ^9^.

## Von Willebrand factor genetics

Long before any genome-wide association studies (GWASs) were performed for VWF levels, a strong link between ABO blood group types and VWF was well established ^10^. More recently, it has been demonstrated that individuals with ABO type O blood have lower VWF levels due to increased clearance of VWF from the circulation compared with individuals with type A or B blood groups. However, the precise mechanisms, such as glycosylation patterns of VWF or glycosylation patterns of multiple clearance receptors, are yet to be fully resolved ^11– 13^. *ABO* blood group haplotypes account for 30% of the genetic variability in plasma VWF, but what about the remaining 70%? To discover new common variants associated with plasma VWF variation, investigators have recently performed large GWASs and linkage studies.

GWAS analyses scan the genomes of study populations looking for strong associations between single-nucleotide variants (single-nucleotide polymorphisms [SNPs]) and the study phenotype. Because SNPs, like other variants in the genome, are inherited in linkage disequilibrium blocks, when strong associations are made between a group of SNPs and a trait, any variant in a linkage disequilibrium block tagged by these SNPs may play a role in the phenotype. Knowing that *ABO* haplotypes are associated with VWF levels and that these haplotypes can be marked by common SNPs, we expect that SNPs marking the common blood type haplotypes in the *ABO* gene would be a strong signal in any adequately powered GWAS for VWF.

## Common variant association studies

The first large GWAS for VWF reported a strong association with common SNPs in or near *ABO*, *VWF*, *STXBP5*, *SCARA5*, *STX2*, *TC2N*, *STAB2*, and *CLEC4M* ^14^. In order to identify these signals, 23,608 individuals from five different study cohorts were analyzed. The signal at *ABO* was by far the strongest, and variants at *VWF* were the second strongest. Taken together, the significant SNPs in this study explained 12.8% of the variation in plasma VWF levels. This number may be confusing, as previous studies had explained more VWF variation at *ABO* alone. This lower number in a vastly larger study population likely relates to the increased pre-analytical variation that the study could not control for, such as some of the environmental factors listed above and heterogeneity introduced by combining several different study cohorts. Despite the smaller-than-expected variation explained in this report, the study was extremely useful to identify potential new genetic loci associated with VWF that were previously undescribed.

Concurrent with the large GWAS described above, our group took a different approach to GWAS for VWF. In order to limit the environmental variables that alter VWF levels, we limited our study to a younger healthy population age (14–35 years) and performed a detailed “environmental” phenotyping survey for every participant. Plasma was processed within one hour of the blood draw, and the coefficient of variation in our VWF antigen assays was less than 4%. Additionally, in order to detect the association with rare variants, which is discussed below, we concentrated on the recruitment of related individuals (siblings). The disadvantage of this approach was the slower recruitment of siblings compared with unrelated individuals and the reduction in power in GWAS analyses where related individuals are included. This study included 1,152 individuals of European ancestry with plasma VWF levels available for GWAS and linkage. The heritability of VWF levels in our study was 64.5%. In meta-analysis with an independent young healthy cohort of 2,310 individuals, our study demonstrated a strong association with common variants at the *ABO* and *VWF* loci. There were no other signals detected in GWAS, suggesting that the power of this study was inadequate to detect other loci with small effect sizes.

In a subsequent study of the same cohort of siblings, we measured VWF propeptide (VWFpp) levels in order to determine whether the variants associated with VWF were operating through synthesis/secretion or clearance mechanisms. VWFpp is synthesized in molar ratios equal to those of VWF but is rapidly cleared from the circulation; VWFpp has a half-life of 2–3 hours, whereas multimeric VWF has a longer half-life of 8–12 hours ^15^. The rapid clearance of VWFpp leads to steady-state plasma VWFpp levels that are largely dependent on synthesis/secretion rates. Therefore, the ratio of VWFpp to VWF is used as a proxy for VWF clearance rates ^16^. In this study, we demonstrated that ABO blood group haplotypes are associated with changes in the VWFpp-to-VWF ratio. Unexpectedly, we also found an association with VWFpp levels and *ABO*, suggesting that the glycosylation patterns dictated by ABO blood groups also alter the clearance of VWFpp. At the *VWF* locus, variants associated with VWF levels were not associated with VWFpp, which implies that they operate through alternative clearance methods ^17^.

The above studies concentrated on the examination of individuals of European ancestry. Two other important GWASs for VWF levels, in African-American populations, have been subsequently published. These studies replicated the strong association at *ABO* with common blood type haplotypes but also identified African ancestry-specific associations at the *VWF* locus ^18, 19^.

## Functional follow-up studies of genome-wide association study signals

It is important to note that GWAS signals do not often identify the specific genetic variant that may be responsible for an association. Oftentimes, functional follow-up studies must make the assumption that the SNP is in linkage disequilibrium with an unidentified functional regulatory or non-synonymous coding variant in a nearby gene. This complicates the mechanistic understanding of gene phenotype associations. Specifically, it is not always straightforward to connect a gene deficiency model of a GWAS locus to the phenotype being studied. The large VWF GWAS identified several new loci with small effect associations with VWF and factor VIII (FVIII) levels that may not be driven by a genetic deficiency of the tagged gene. Of these GWAS loci, *STXBP5* and *CLEC4M* are the subject of recently published functional studies. STXBP5 is a member of the SNARE complex of proteins that regulate the secretion of proteins in the post-Golgi compartment. STXBP5 is thought to disrupt the formation of a functional complex of STX1, SNAP25, and VAMP2, thereby inhibiting the secretion of proteins. Knockdown of STXBP5 in human umbilical vein endothelial cells leads to increased VWF and P-selectin secretion, while mice deficient in *Stxbp5* have increased plasma VWF levels ^20^. *CLEC4M* encodes a C-type leptin receptor that may function as a scavenger for several plasma glycoproteins. In mammalian cell culture models, CLEC4M binds and internalizes VWF, suggesting that the GWAS signal may be generated by variants with different VWF clearance kinetics ^21^.

Many other studies have made functional connections between plasma VWF levels and other proteins that have not been identified in GWASs. Does this mean that these proteins have no role in VWF biology? Probably not. The absence of a GWAS signal simply reduces the possibility that common genetic variants in genes encoding these proteins play a role in plasma-level variation. Absence of GWAS signal does not eliminate the possibility that rare or “private” variants in these genes play important roles regulating VWF levels in the human population. An excellent example of this limit for GWAS interpretation is with VWF and low-density lipoprotein receptor-related protein 1 (LRP1). Plausible mechanistic studies have demonstrated a potential role for LRP1 as a clearance receptor for plasma VWF, but no signal was detected for *LRP1* in GWASs ^22, 23^. This strongly rejects the hypothesis that common genetic variants in *LRP1* are associated with plasma VWF, but it does not eliminate the possibility that loss-of-function mutations in *LRP1* would alter the clearance kinetics of VWF *in vivo*. Likewise, compensation for loss of a clearance receptor by an alternate receptor could mask a GWAS signal. Other VWF clearance receptors for human VWF, such as the macrophage galactose-type lectin receptor, stabilin-2, and CLEC4M, have been identified. If LRP1 does play a functional role in the clearance of plasma VWF, we expect that different, rare, damaging mutations in *LRP1* are more likely to occur in individuals with higher VWF levels than those without high VWF levels. This type of study requires DNA sequencing in large numbers of individuals and has not been reported to date.

## Linkage studies

Linkage studies differ from GWASs because the former require a study cohort to have family structure. Linkage analyses use genotype patterns to assemble haplotype blocks (linear stretches of shared variant patterns inherited as an ancestral block) in order to define areas of allele sharing within families that are associated with a phenotype. Linkage studies have long been used to map genes causing highly penetrant Mendelian diseases but have had less success in complex genetic traits. An earlier important family study of VWF genotyped 398 individuals in 21 Spanish pedigrees and searched for linkage with VWF levels ^24^. Not surprisingly, they found linkage to the *ABO* locus, but, perhaps more surprisingly, they did not find linkage at the *VWF* gene locus on chromosome 12. Our group used a cohort of 1,284 individuals in 561 sibships from the two healthy cohorts described above and identified signals at *ABO.* Interestingly, like the Spanish study, we did not find a signal at *VWF.* This suggests that although common variants at the *VWF* locus are associated with plasma VWF variation, there were not enough variants of large effect size in these studies to contribute to a significant linkage signal ^3, 24^. The lack of signal at *VWF* should be confirmed through larger DNA sequencing studies of VWF variation which address the contribution of rare variants. Although no linkage signal was identified at *VWF*, our linkage analysis detected a strong linkage signal on chromosome 2q12-2p13 with an effect size similar to that of *ABO* (19.2% and 24.5%, respectively). This large linkage signal was previously identified in a family study of coagulation FVIII levels (which are largely determined by VWF levels) but had not been previously linked to plasma VWF ^25^. This linkage interval was not detected in GWASs, suggesting that the signal was generated by several different alleles, individually below the detection frequency in GWASs but collectively significant by linkage. We hypothesize that sequencing studies in these healthy siblings should identify a locus in the linkage interval where clusters of rare variants will, in aggregate, associate with VWF level variation.

## Rare variant studies

The statistical power to identify single-variant associations is dependent on several variables, including the allele frequency and the effect size of the variant on the trait. This explains why the *ABO* locus gives such a powerful signal in VWF GWASs, as the O allele has a frequency of about 40% and an effect size of about 25%. Likewise, when considering rare variants (loosely defined as variants with minor allele frequencies below 1%), the power to detect significant associations is reduced unless the number of individuals studied is dramatically increased. The best current method to identify genome-wide rare variants in a population is through the use of next-generation DNA sequencing. However, the initial costs of this approach were prohibitive, leading to the use of specialized genotyping chips focusing on non-synonymous variants (those that change the protein code of genes) that were previously characterized in large population sequencing studies (“exome chips”). Another technique was to employ imputed genotype datasets which predict the presence of variants based on the haplotype patterns present in the genotyped portion of the genome. Once rare variants have been identified in a study population, other techniques have been used to overcome the statistical powering barrier. One promising method is the use of aggregate tests of association at the gene level, sometimes referred to as gene collapsing tests or mutation burden tests. These analyses assume that loss of function in a gene may contribute to the genetic determination of a trait. Because many different variants can cause loss of function, such as nonsense, frameshift, splice site, and damaging missense mutations, tests that aggregate qualifying variants in a gene can increase statistical power. Other tests attempt to account for the effect direction of variants, which may not be equal for every variant in a gene. Rare variant association testing was nicely reviewed by Auer and Lettre ^26^.

Recently, the same group that published the large VWF GWAS reported on the use of “exome chips” and imputation to look for rare variant associations in a large population of unrelated individuals ^27^. This study analyzed plasma VWF levels in 76,000 individuals and identified a single variant in *STAB2* that was associated with increased VWF (and FVIII) concentrations. In this study, *STAB2* (encodes stabilin-2, a sinusoidal endothelially expressed scavenger receptor) was also significant in gene-based tests. It is hypothesized that loss of function of stabilin-2 may reduce the clearance of VWF, leading to longer plasma half-lives and higher plasma levels.

## Role of von Willebrand factor-associated variants in disease

Identification of multiple loci harboring common SNPs associated with VWF levels spurred the investigation of these loci in individuals with VWD and venous thrombosis. Elevated VWF is a well-described risk factor for VTE, and this likely explains why ABO blood group haplotypes are also associated with VTE risk. A recent study looked for connections between other VWF GWAS SNPs and VTE risk. In this study, 656 women with incident VTE and 710 controls were genotyped for the top SNPs at seven loci identified in VWF GWAS. SNPs at both *VWF* and *STXBP5* were associated with VTE risk ^28^. Interestingly, the SNPs tagging *STXBP5* and *VWF* were not replicated at a genome-wide significant level in a much larger GWAS of 7,507 VTE cases and 52,632 controls, suggesting that these common variants do not play a major role in VTE risk ^29^.

At the other end of the VWF distribution, several studies have looked at the role of VWF-associated variants in VWD and found that individuals with VWD type 1 are more likely to have *ABO* type O blood group haplotypes, especially those individuals who have milder forms of VWD type I ^30, 31^. More recent studies have tested the association with VWF GWAS loci and VWD. A study of 364 Dutch individuals with VWD type I showed significant associations with common variants in *STXBP5* and *CLEC4M* ^32^. As it is difficult to accrue patients with VWD into a large genetic study, an alternative approach to this question is to compare individuals with low VWF levels with those with high levels and perform a case control GWAS. When examining plasma VWF levels in 31,149 European ancestry participants and performing a GWAS between the lowest fifth percentile of VWF levels and the top 5% of the cohort, investigators found significant signals at *ABO*, *VWF*, *STXPB5*, *STAB2*, and *UFM1* ^33^. These results do not confirm the role of these variants in symptomatic bleeding due to low VWF levels as previous studies have suggested ^34^, but they do suggest that an adequately powered study of VWD would confirm the genome-wide significance and role of these common variants in VWD.

## Unanswered questions

To date, investigators have taken advantage of genome-wide SNP genotyping in European and African-American populations to determine the range of common SNPs associated with VWF variation. In doing so, they have identified new loci that were previously undescribed in VWF biology. These studies have led to interesting follow-up studies to determine the functional mechanisms behind these genetic associations. These functional studies need to be further explored, as questions remain about the mechanistic role of many of these VWF-associated loci. However, the overall small effect size of the signals at GWAS loci and the difficulty identifying the causative genetic variants from GWAS remain a significant challenge for follow-up studies.

Rare variants have yet to be fully explored for their role in VWF regulation. Initial reports using exome chips and imputation are still limited by their ability to accurately predict very low-frequency variants. Rare variant studies require a commitment to whole genome sequencing of a large number of individuals with high-quality phenotyping to overcome the limitations of statistical power. The National Heart, Lung, and Blood Institute’s Trans-Omics for Precision Medicine (TOPMed) program is a good example of the National Institutes of Health’s commitment to these scientific questions ^35^. Investigators are hopeful that rare variant discoveries may be easier to translate into a biologic mechanism, as they are predicted to have higher effect sizes and more likely to be causal variants compared with common SNPs.

More recently, Mendelian randomization studies have become an additional tool to connect the genetic regulation of quantitative traits (such as VWF or FVIII levels) to other diseases. These studies could be used to correlate elevated plasma VWF levels to the risk for more complex medical diseases such as VWD, VTE, ischemic stroke, coronary artery disease, and thrombotic thrombocytopenic purpura where previous studies have suggested a possible biologic connection ^36^.

In summary, efforts to uncover a comprehensive set of genetic determinants of VWF variation should lead to a better mechanistic understanding of VWF biology and complex medical disorders where this critical plasma protein plays a central role.
